# Crystal structure of hexa­aqua­dichlorido­ytterbium(III) chloride

**DOI:** 10.1107/S2056989015008488

**Published:** 2015-05-07

**Authors:** Kevin M. Knopf, Guy Crundwell, Barry L. Westcott

**Affiliations:** a Department of Chemistry & Biochemistry, Central Connecticut State University, New Britain, CT 06053, USA

**Keywords:** crystal structure, ytterbium(III), chloride, hydrogen bonding

## Abstract

The crystal structure of the title compound, [YbCl_2_(H_2_O)_6_]Cl, was determined at 110 K. Samples were obtained from evaporated aceto­nitrile solutions containing the title compound, which consists of a [YbCl_2_(H_2_O)_6_]^+^ cation and a Cl^−^ anion. The cations in the title compound sit on a twofold axis and form O—H⋯Cl hydrogen bonds with the nearby Cl^−^ anion. The coordination geometry around the metal centre forms a distorted square anti­prism. The ytterbium complex is isotypic with the europium complex [Tambrornino *et al.* (2014 ▸). *Acta Cryst.* E**70**, i27].

## Related literature   

The ytterbium complex is isotypic with the europium complex, the redetermined structure of which was published recently (Tambrornino *et al.* 2014 ▸) which was in turn similar to studies of other lanthanoid chloride hydrates (Marezio *et al.*, 1961 ▸).

## Experimental   

### Crystal data  

[YbCl_2_(H_2_O)_6_]Cl
*M* *_r_* = 387.49
Monoclinic, 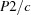

*a* = 7.8158 (11) Å
*b* = 6.4651 (3) Å
*c* = 12.7250 (18) Å
β = 131.45 (2)°
*V* = 481.92 (16) Å^3^
*Z* = 2
Mo *K*α radiation
μ = 10.52 mm^−1^
*T* = 110 K
0.24 × 0.18 × 0.17 mm

### Data collection  

Oxford Diffraction Xcalibur Sapphire3 diffractometer
Absorption correction: multi-scan (*CrysAlis PRO*; Oxford Diffraction, 2009 ▸) *T*_min_ = 0.187, *T*_max_ = 0.268
12358 measured reflections
1806 independent reflections
1762 reflections with *I* > 2σ(*I*)
*R*_int_ = 0.042

### Refinement  

*R*[*F* ^2^ > 2σ(*F* ^2^)] = 0.018
*wR*(*F* ^2^) = 0.041
*S* = 1.12
1806 reflections
51 parameters
H-atom parameters constrained
Δρ_max_ = 0.91 e Å^−3^
Δρ_min_ = −0.96 e Å^−3^

### 

Data collection: *CrysAlis CCD* (Oxford Diffraction, 2009 ▸); cell refinement: *CrysAlis RED* (Oxford Diffraction, 2009 ▸); data reduction: *CrysAlis RED*; program(s) used to solve structure: *SHELXS2014* (Sheldrick, 2008 ▸); program(s) used to refine structure: *SHELXL2014* (Sheldrick, 2015 ▸); molecular graphics: *PLATON* (Spek, 2009 ▸) and *ORTEP-3 for Windows* (Farrugia, 2012 ▸); software used to prepare material for publication: *SHELXL2014*.

## Supplementary Material

Crystal structure: contains datablock(s) I. DOI: 10.1107/S2056989015008488/br2249sup1.cif

Structure factors: contains datablock(s) I. DOI: 10.1107/S2056989015008488/br2249Isup2.hkl

**Fig. 1.**
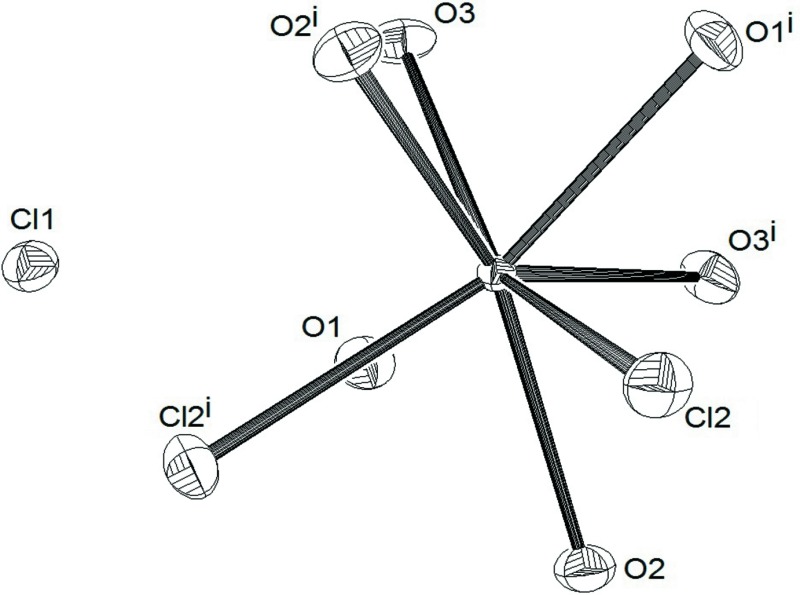
A view of the title compound (Farrugia, 2012). Displacement ellipsoids are drawn at the 50% probability level. H atoms have been omitted for clarity.

. DOI: 10.1107/S2056989015008488/br2249fig1.tif
A view of the title compound (Farrugia, 2012). Displacement ellipsoids are drawn at the 50% probability level. H atoms have been omitted for clarity.

CCDC reference: 1062504

Additional supporting information:  crystallographic information; 3D view; checkCIF report

## Figures and Tables

**Table 1:** Hydrogen-bond geometry (, )

*D*H*A*	*D*H	H*A*	*D* *A*	*D*H*A*
O1H1*A*Cl2^i^	0.91	2.37	3.2499(19)	163
O1H1*B*Cl1^ii^	0.91	2.50	3.171(2)	131
O2H2*A*Cl1^iii^	0.87	2.36	3.1460(18)	150
O2H2*B*Cl2^iv^	0.87	2.38	3.1806(18)	154
O3H3*A*Cl2^v^	0.88	2.33	3.179(2)	163
O3H3*B*Cl1^vi^	0.88	2.48	3.1758(19)	136

Symmetry codes: (i) 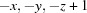; (ii) 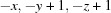; (iii) 
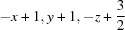
; (iv) 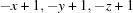; (v) 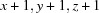; (vi) 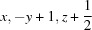.

## supplementary crystallographic information

### S1. Comment

Samples gathered from the mother liquor were coated with mineral oil prior to mounting to reduce sample decay. The ytterbium complex is isomorphous with a recently published redetermination of a europium complex (Tambrornino, *et al.* 2014) which was in turn similar to studies of other lanthanoid chloride hydrates (Marezio, *et al.* 1961).

Crystals of ytterbium(III) chloride hexahydrate consist of a [YbCl_2_(H_2_O)_6_]^1+^ cation and a chlorine anion. The covalent nature of the lanthanoid +3 cations are not surprising given their high charge density. An *ORTEP* of the title compound is shown in Fig. 1.

### S2. Experimental

In 40.0 ml of acetonitrile, 0.1945 grams (0.5019 mmol) of ytterbium(III) chloride hexahydrate was added to 0.1000 grams (0.5020 mmol) of di-2-pyridyl ketone oxime (dpko) with the hopes of synthesizing a Yb-dpko complex. The mixture was heated to dissolve the solids. Upon cooling and subsequent evaporation, small colorless crystals of the title compound were isolated. The metal chloride was purchased from Strem chemicals (99.9% purity) whereas the dpko was purchased from Sigma-Aldrich (99.9%). Both were used without additional purification.

### S3. Refinement

H atoms were included and were allowed to refine to ideal O—H distances based upon geometric considerations. Thermal parameters for all H atoms were included in the refinement in riding motion approximation with *U*_iso_ = 1.5*U*_eq_ of the carrier atom.

### Crystal data

**Table:**

[YbCl_2_(H_2_O)_6_]Cl	*D*_x_ = 2.671 Mg m^−^^3^
*M**_r_* = 387.49	Melting point: 350 K
Monoclinic, *P*2/*c*	Mo *K*α radiation, λ = 0.71073 Å
*a* = 7.8158 (11) Å	Cell parameters from 7486 reflections
*b* = 6.4651 (3) Å	θ = 4.9–33.8°
*c* = 12.7250 (18) Å	µ = 10.52 mm^−^^1^
β = 131.45 (2)°	*T* = 110 K
*V* = 481.92 (16) Å^3^	Block, light pink
*Z* = 2	0.24 × 0.18 × 0.17 mm
*F*(000) = 362	

### Data collection

**Table:**

Oxford Diffraction Xcalibur Sapphire3 diffractometer	1806 independent reflections
Radiation source: fine-focus sealed tube	1762 reflections with *I* > 2σ(*I*)
Graphite monochromator	*R*_int_ = 0.042
Detector resolution: 16.1790 pixels mm^-1^	θ_max_ = 33.7°, θ_min_ = 4.3°
ω scans	*h* = −11→12
Absorption correction: multi-scan (*CrysAlis PRO*; Oxford Diffraction, 2009)	*k* = −9→9
*T*_min_ = 0.187, *T*_max_ = 0.268	*l* = −19→19
12358 measured reflections	

### Refinement

**Table:**

Refinement on *F*^2^	Hydrogen site location: difference Fourier map
Least-squares matrix: full	H-atom parameters constrained
*R*[*F*^2^ > 2σ(*F*^2^)] = 0.018	*w* = 1/[σ^2^(*F*_o_^2^) + (0.0207*P*)^2^] where *P* = (*F*_o_^2^ + 2*F*_c_^2^)/3
*wR*(*F*^2^) = 0.041	(Δ/σ)_max_ = 0.001
*S* = 1.12	Δρ_max_ = 0.91 e Å^−^^3^
1806 reflections	Δρ_min_ = −0.96 e Å^−^^3^
51 parameters	Extinction correction: *SHELXL2014* (Sheldrick, 2015), Fc^*^=kFc[1+0.001xFc^2^λ^3^/sin(2θ)]^-1/4^
0 restraints	Extinction coefficient: 0.0602 (13)

### Special details

**Table:**

Experimental. Sample was covered in mineral oil prior to mounting in cryo stream.Hydrogen atoms were included and were allowed to refine to ideal O—H distances based upon geometric considerations. Thermal parameters for all H atoms were included in the refinement in riding motion approximation with *U*_iso_ = 1.5*U*_eq_ of the carrier atom.CrysAlisPro, Oxford Diffraction Ltd., Version 1.171.33.52 (release 06-11-2009 CrysAlis171 .NET) Empirical absorption correction using spherical harmonics, implemented in SCALE3 ABSPACK scaling algorithm (Oxford Diffraction (2009).
Geometry. All esds (except the esd in the dihedral angle between two l.s. planes) are estimated using the full covariance matrix. The cell esds are taken into account individually in the estimation of esds in distances, angles and torsion angles; correlations between esds in cell parameters are only used when they are defined by crystal symmetry. An approximate (isotropic) treatment of cell esds is used for estimating esds involving l.s. planes.

### Fractional atomic coordinates and isotropic or equivalent isotropic displacement parameters (Å^2^)

**Table:**

	*x*	*y*	*z*	*U*_iso_*/*U*_eq_	
Yb1	0.5000	0.65776 (2)	0.7500	0.01599 (6)	
Cl1	0.32165 (12)	0.34336 (8)	0.56141 (7)	0.02594 (12)	
Cl2	0.0000	−0.12652 (14)	0.2500	0.02883 (16)	
O1	0.1817 (3)	0.5542 (3)	0.71889 (19)	0.0266 (3)	
H1A	0.1626	0.4279	0.7416	0.040*	
H1B	0.0569	0.6336	0.6823	0.040*	
O2	0.7626 (3)	0.9242 (3)	0.85341 (19)	0.0276 (3)	
H2A	0.7791	1.0181	0.9087	0.041*	
H2B	0.8611	0.9467	0.8434	0.041*	
O3	0.5420 (3)	0.8002 (3)	0.93497 (19)	0.0273 (3)	
H3A	0.6709	0.8455	1.0145	0.041*	
H3B	0.4315	0.8170	0.9361	0.041*	

### Atomic displacement parameters (Å^2^)

**Table:**

	*U*^11^	*U*^22^	*U*^33^	*U*^12^	*U*^13^	*U*^23^
Yb1	0.01594 (8)	0.01718 (8)	0.01703 (8)	0.000	0.01184 (6)	0.000
Cl1	0.0283 (3)	0.0258 (3)	0.0251 (3)	−0.00427 (18)	0.0183 (2)	−0.00488 (18)
Cl2	0.0271 (4)	0.0336 (4)	0.0299 (4)	0.000	0.0206 (4)	0.000
O1	0.0229 (8)	0.0278 (8)	0.0343 (9)	−0.0001 (6)	0.0212 (8)	0.0043 (7)
O2	0.0293 (9)	0.0262 (8)	0.0359 (9)	−0.0096 (7)	0.0253 (8)	−0.0098 (7)
O3	0.0310 (9)	0.0331 (8)	0.0246 (8)	−0.0050 (7)	0.0213 (8)	−0.0061 (7)

### Geometric parameters (Å, º)

**Table:**

Yb1—O2^i^	2.3101 (17)	Yb1—O1^i^	2.3433 (16)
Yb1—O2	2.3101 (17)	Yb1—O1	2.3434 (17)
Yb1—O3^i^	2.3392 (17)	Yb1—Cl1^i^	2.7211 (7)
Yb1—O3	2.3392 (17)	Yb1—Cl1	2.7212 (7)
			
O2^i^—Yb1—O2	83.56 (10)	O1^i^—Yb1—Cl1^i^	76.75 (5)
O2^i^—Yb1—O3^i^	69.72 (6)	O1—Yb1—Cl1^i^	78.60 (5)
O2—Yb1—O3^i^	76.09 (7)	O2^i^—Yb1—Cl1	108.14 (6)
O2^i^—Yb1—O3	76.09 (7)	O2—Yb1—Cl1	143.38 (5)
O2—Yb1—O3	69.72 (6)	O3^i^—Yb1—Cl1	75.99 (5)
O3^i^—Yb1—O3	133.64 (9)	O3—Yb1—Cl1	146.11 (5)
O2^i^—Yb1—O1^i^	138.85 (6)	O1^i^—Yb1—Cl1	78.60 (5)
O2—Yb1—O1^i^	70.92 (6)	O1—Yb1—Cl1	76.75 (5)
O3^i^—Yb1—O1^i^	73.06 (7)	Cl1^i^—Yb1—Cl1	83.34 (3)
O3—Yb1—O1^i^	121.09 (7)	Yb1—O1—H1A	125.5
O2^i^—Yb1—O1	70.92 (6)	Yb1—O1—H1B	125.7
O2—Yb1—O1	138.85 (6)	H1A—O1—H1B	108.8
O3^i^—Yb1—O1	121.09 (7)	Yb1—O2—H2A	125.4
O3—Yb1—O1	73.06 (7)	Yb1—O2—H2B	125.4
O1^i^—Yb1—O1	146.79 (9)	H2A—O2—H2B	109.2
O2^i^—Yb1—Cl1^i^	143.38 (5)	Yb1—O3—H3A	125.4
O2—Yb1—Cl1^i^	108.15 (6)	Yb1—O3—H3B	125.4
O3^i^—Yb1—Cl1^i^	146.11 (5)	H3A—O3—H3B	109.2
O3—Yb1—Cl1^i^	75.99 (5)		

Symmetry code: (i) −*x*+1, *y*, −*z*+3/2.

### Hydrogen-bond geometry (Å, º)

**Table:**

*D*—H···*A*	*D*—H	H···*A*	*D*···*A*	*D*—H···*A*
O1—H1*A*···Cl2^ii^	0.91	2.37	3.2499 (19)	163
O1—H1*B*···Cl1^iii^	0.91	2.50	3.171 (2)	131
O2—H2*A*···Cl1^iv^	0.87	2.36	3.1460 (18)	150
O2—H2*B*···Cl2^v^	0.87	2.38	3.1806 (18)	154
O3—H3*A*···Cl2^vi^	0.88	2.33	3.179 (2)	163
O3—H3*B*···Cl1^vii^	0.88	2.48	3.1758 (19)	136

Symmetry codes: (ii) −*x*, −*y*, −*z*+1; (iii) −*x*, −*y*+1, −*z*+1; (iv) −*x*+1, *y*+1, −*z*+3/2; (v) −*x*+1, −*y*+1, −*z*+1; (vi) *x*+1, *y*+1, *z*+1; (vii) *x*, −*y*+1, *z*+1/2.

## References

[bb1] Farrugia, L. J. (2012). *J. Appl. Cryst.* **45**, 849–854.

[bb2] Marezio, M., Plettinger, H. A. & Zachariasen, W. H. (1961). *Acta Cryst.* **14**, 234–236.

[bb3] Oxford Diffraction (2009). *CrysAlis CCD*,*CrysAlis PRO* and *CrysAlis RED*. Oxford Diffraction Ltd, Abington, England.

[bb4] Sheldrick, G. M. (2008). *Acta Cryst.* A**64**, 112–122. 10.1107/S0108767307043930 18156677

[bb5] Sheldrick, G. M. (2015). *Acta Cryst.* C**71**, 3–8.

[bb6] Spek, A. L. (2009). *Acta Cryst.* D**65**, 148–155. 10.1107/S090744490804362X PMC2631630 19171970

[bb7] Tambornino, F., Bielec, P. & Hoch, C. (2014). *Acta Cryst.* E**70**, i27. 10.1107/S1600536814010307 PMC4051107 24940187

