# Supplementary figures and images for: Crystal structure of hexa­aqua­dichlorido­ytterbium(III) chloride
Source: Acta Crystallogr E Crystallogr Commun. 2015 May 7;71(Pt 6):i5. doi: 10.1107/S2056989015008488 (PMC4459311; doi:10.1107/S2056989015008488)

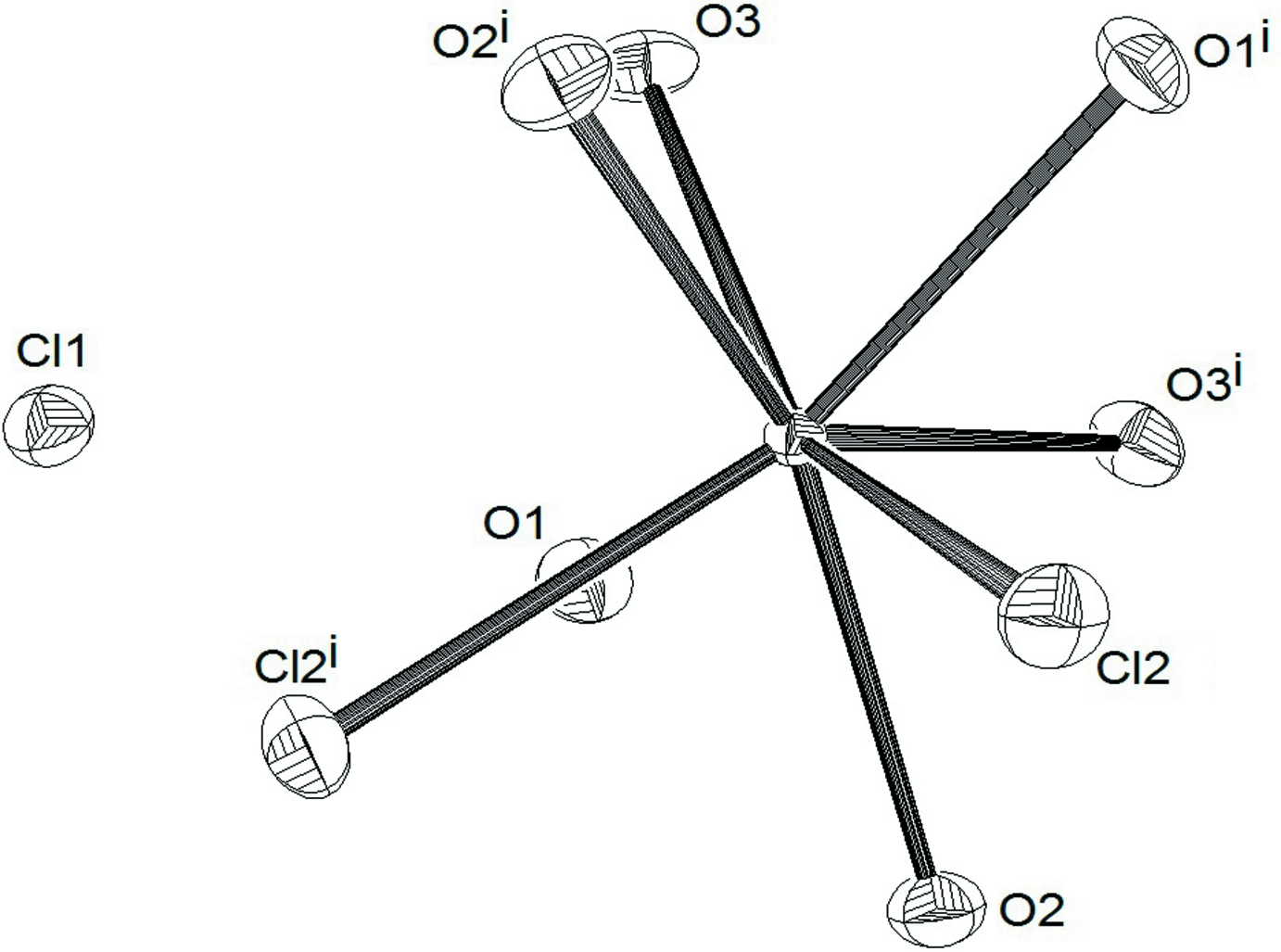

Supplement: Supplementary file 3 [file e-71-000i5-fig1.tif]
